# Factors associated with retention on pre-exposure prophylaxis among female sex workers in Kigali, Rwanda

**DOI:** 10.1371/journal.pgph.0002524

**Published:** 2023-11-06

**Authors:** Sezi Mubezi, Gallican N. Rwibasira, Jeanne Uwineza, Jean de Dieu Kayisinga, Manasseh G. Wandera, Samuel S. Malamba, Chrispus Mayora, Joseph K. B. Matovu

**Affiliations:** 1 Health Program Unit, Society for Family Health, Kigali, Rwanda; 2 Makerere University School of Public Health, Kampala, Uganda; 3 Rwanda Biomedical Center, Ministry of Health, Kigali, Rwanda; 4 Health Research and Evaluation Consultant, Kampala, Uganda; 5 Department of Community and Public Health, Faculty of Health Sciences, Busitema University, Mbale, Uganda; PLOS: Public Library of Science, UNITED STATES

## Abstract

Pre-Exposure Prophylaxis (PrEP) is recommended as an additional HIV prevention measure for persons at substantial risk of HIV acquisition. Although uptake of PrEP among female sex workers (FSW) has increased, retention remains low, resulting in suboptimal benefits. This study aimed at determining PrEP retention rates and associated factors among FSW in Kigali, Rwanda. We retrospectively studied records of 309 FSW abstracted from five (5) health centers for the period between April-June 2020 and April-June 2021. PrEP retention was defined as presenting for a scheduled follow-up visit. We used Kaplan-Meier survival analysis to estimate survival probabilities at months 1,3,6,9, and 12 post-PrEP initiation and Cox regression to determine factors associated with 12-month PrEP retention. Data was analyzed using STATA (version 14.0). Out of 309 FSW whose records were reviewed, data for 268 (87%) were complete. One half (50%, n = 133) of the respondents were aged 25–34 years; slightly more than half (52%, n = 136) were single; nearly three-quarters (73%, n = 196) had completed primary school; majority (88%, n = 236) lived alone; while 69% (n = 184) had no formal employment besides sex work. PrEP dropout rates were 228, 65, 29, 49, and 36 per 100-persons years at months 1, 3, 6, 9 and 12 respectively, with 81%, 72%, 67%, 59% and 53% of FSW that started PrEP retained at these time periods. Multivariable Cox regression revealed that compared to FSW opposed to additional children, the desire to have two or more children (adjusted Hazard Ratio [aHR] = 1.654; 95% Confidential Interval [95%CI]: 1.008, 2.713); and using hormonal (aHR = 2.091, 95%CI: 1.181, 3.702) or no method of contraception other than condoms (aHR = 2.036, 95%CI: 1.006, 4.119) were factors positively associated with PrEP retention. Conversely, compared to consistent condom-use, not using (aHR = 0.329; 95%CI: 0.149, 0.726) or inconsistently using condoms (aHR = 0.413; 95%CI: 0.228, 0.749), and accessing PrEP from ultra-urban clinics (aHR = 0.290; 95%CI: 0.183, 0.458) compared to clinics in the outskirts of the city, were factors negatively associated with PrEP retention. The study found a continuous decline in PrEP retention among FSW with slightly more than half retained at 12 months. To improve outcomes, PrEP retention monitoring should target FSW enrolled in ultra-urban clinics and those not or inconsistently using condoms.

## Introduction

In 2021, it was reported that key populations (KPs) and their sexual partners accounted for 70% of the 1.5 million new HIV (Human Immunodeficiency Virus) infections globally [1]. In the same period, female sex workers (FSW) accounted for 12% of new HIV infections globally [1]. It is widely reported that FSW have a high risk of acquiring HIV infection ranging from 12–30 fold compared to other females in the general population [1–6]. Given these figures, FSW and other KPs, were singled out in the 2021 political declaration on Acquired Immunodeficiency Syndrome (AIDS) as being at heightened risk of HIV acquisition requiring critical interventions to stem the tide [6].

Pre-Exposure Prophylaxis (PrEP) is an HIV prevention method in which HIV negative individuals with substantial risk of HIV acquisition take a daily pill of tenofovir/emtricitabine (TDF/FTC) commonly known as Truvada or tenofovir/lamivudine (TDF/3TC) to prevent HIV infection. The effectiveness of PrEP for HIV prevention has been demonstrated in several studies including iPrEx (Pre-Exposure Initiative), the Partners PrEP study, the Bangkok Tenofovir study, Strand study, among others [7–10]. Protection levels varied from 44% - 99% depending on the study and levels of adherence achieved [11]. Based on this, PrEP was recommended by the World Health Organization (WHO) for persons at substantial risk of HIV acquisition of which FSW fit the characterization [12–14].

The uptake of PrEP has been high in most settings where PrEP has been made available to FSW. Demonstration projects in Benin and South Africa showed a PrEP uptake rate among FSW of 88.3% and 98% respectively [15, 16]. In India, a community-led HIV program for FSW demonstrated an uptake rate of 80% among those eligible [17]. A PrEP uptake rate of 93.3% among FSW was demonstrated in South-Central Uganda in the districts of Rakai, Masaka, Kyotera, and Lyantonde [18]. High PrEP uptake among FSW or generally is driven by many factors including: a perception of high HIV risk when engaging with a partner who is HIV positive, or one whose HIV status is unknown, or when it is challenging to consistently use condoms for every sexual encounter as might be the case with clients paying more money [19, 20]. Given this, uptake of PrEP among FSW is thus not a major issue and is projected to remain high provided that FSW are helped to accurately assess their own HIV risk and PrEP made available to them as an alternative HIV prevention method.

Once anyone initiates PrEP, they need to adhere to the medication to optimize its benefits. This is especially critical for FSW given that women require near perfect adherence, in any case not less than 85%, to reach and maintain protective PrEP therapeutic levels in cervico-vaginal tissues [21, 22]. In fact, two PrEP trials (Preexposure Prophylaxis Trial for HIV Prevention among African Women–FEM-PrEP and Vaginal and Oral Interventions to Control the Epidemic–VOICE) were considered futile because of low adherence levels among participants [23, 24].

Critical in achieving good adherence levels is the aspect of retention; given that those clients that are retained on PrEP will get the drug refills, laboratory monitoring, as well as adherence counseling required [25]. However, retention rates among FSW receiving PrEP have been reported to be low. The TAPS (Treatment And Prevention for female Sex workers) project in South Africa demonstrated 12-months retention of 22% for FSW that had initiated PrEP [16]. In the SAPPH-IRe (Sisters Antiretroviral Programme for Prevention of HIV, an Integrated Response) trial in Zimbabwe, FSW took PrEP for about 4 months on average [26]. The demonstration project in Benin recorded a 12 month retention of 47.3%, while the one in Kenya recorded retention rates of 40, 26, and 14% at months 1, 3, and 6 respectively [27, 28]. Even in the program mode, retention of FSW on PrEP remains low as seen in South-Central Uganda [18].

In Rwanda, the incidence rate of HIV cases reported in 2019 among FSW was 3.5 per 100 person years which is higher than the general population incidence rate of 0.27 per 100 person years [29]. Some of the factors associated with increased sexual risk that may eventually contribute to high HIV incidence rates among FSW in Rwanda include inconsistent condom use, having sex while intoxicated with alcohol, physical and sexual violence, high prevalence of STIs, low level of comprehensive HIV knowledge, among other factors [29, 30]. These factors present a strong case for the integration of PrEP into the HIV prevention mix for FSW. Since 2018, Rwanda recommends PrEP for KPs in tandem with the Centers for Disease Control and Prevention (CDC) guidelines, WHO guidelines, and the Rwanda HIV and AIDS national strategic plan [31–33]. PrEP is projected to reduce HIV incidence in Rwanda by 1.28% by 2027 [29].

Implementation of the PrEP intervention among FSW and other KPs in Rwanda started in April 2019. Program data from society for Family Health (SFH) Rwanda showed that out of 33,542 FSW, 5,169 (15%) had initiated PrEP by 30^th^ September 2021: the initiation rate being low due to the fact the program was still at a pilot phase at that time. Additionally, PrEP retention rates were not known but suspected to be low like in other jurisdictions. This study thus sought to determine PrEP retention rates and the factors associated with retention on PrEP among FSW in Kigali with a view of making proposals to refine strategies aimed at optimizing retention on PrEP and improve outcomes.

## Materials and methods

### Study design and setting

This was a retrospective cohort study of PrEP retention outcomes for FSW who were enrolled on the key population (KP) program implemented by Society for Family Health (SFH) Rwanda. The KP program implementation is supported by the U.S. President’s Emergency Plan for AIDS Relief (PEPFAR) through the US Centers for Disease Control and Prevention (CDC)–Rwanda. The KP program aims at reducing HIV incidence among KPs through provision of targeted services such as HIV testing services (HTS), linkage of HIV positive KPs to Antiretroviral therapy (ART), distribution of HIV self-test kits, risk reduction counseling, post-exposure prophylaxis (PEP), and PrEP. The study was conducted at 5 health centers of Busanza, Kabusunzu, Kacyiru, Kinyinya, and Masaka that are in the 3 districts of Kigali namely: Nyarugenge, Kicukiro, and Gasabo.

### Participants

The PrEP program that started in Rwanda in April 2019 is being implemented in a real-world setting. This retrospective study considered FSW that were initiated on PrEP in the quarter of April to June 2020 and followed for a 12-month period ending in April-June 2021. A FSW enrolled on the study had to be aged 18 years and above. All FSW that were formerly transferred from the participating health centers during the study period were excluded from the study.

### Sample size

Using a single rate precision method for sample size $(n)=\frac{\mu }{e^{2}}$ with an assumed 12-month PrEP retention of 60%, standard error of 0.05, and factoring in 20% missing participants, a sample size of 300 clients was determined as adequate to estimate the PrEP retention rates over the study period [34]. Using the Power Analysis and Sample Size (PASS) software, the sample of 300 was sufficient to detect factors associated with retention achieving a power of 90% or greater in detecting an additional retention rate in the comparison category of 0.16 or higher when the significance level is 0.05.

### Measurement of variables

Retention was the outcome in this study and was categorized as a binary variable coded one (1) for a FSW that presented for their scheduled follow-up visits for PrEP refills at months 1, 3, 6, 9, and 12. A ±14-day window was allowed for those who might have missed their scheduled visit date or came earlier than their scheduled visits. A client that did not return for their scheduled follow-up visits after the 14-day window was coded as zero (0) and determined as not retained on PrEP and dropped. Retention was considered at first instance meaning that a client who missed visit 3 and returned at visit 6, was considered as dropped at visit 3 and not considered for other subsequent visits (in this case, we ignored visit 6). The predictor variables were determined as the factors associated with PrEP retention, captured in a data abstraction form, using data collected during the PrEP eligibility assessment and follow-up visits. Demographic characteristics of the respondents were also captured. During the follow-up visits, FSW were provided with adherence counseling, HIV-retesting, HIV risk assessment, STI screening and treatment, and laboratory creatinine assessment. FSW that remained HIV negative with normal creatinine levels were refilled with a 2-month refill of Truvada (FTC 200mg and TDF 300mg) if they came for month 1 visit, or a 3-month refill for subsequent visits after month-1.

### Data handling and statistical analysis

DATIM (Design and Analysis Toolkit for Inventory and Monitoring—version 17.1), a reporting system used by PEPFAR, was used to determine the number of FSW reported as enrolled on PrEP from five health centers in the Rwanda HIV program. Data were abstracted by trained research assistant using a digitized abstraction developed with the ArcGIS survey123 system that was downloadable on password-protect tablets. Data abstracted was for two similar time-periods in two consecutive years: April-June 2020 and April-June 2021. The time-period April–June 2020 was designated as the PrEP initiation period while the subsequent time-period (April-June 2021) was designated as the retention period. All data were relayed to a protected server at SFH offices which automatically triggered daily deletion of all data on the tablet upon synchronization at 23h 59min. Data from the server were then downloaded, coded, cleaned, and uploaded into a statistical analysis package (STATA 14.0). Data were collected for a 2-weeks period starting from 17^th^ to 31^st^ January 2022. Authors did not have access to information that could identify individual participants during or after data collection.

Descriptive statistics for the demographic and predictor variables were summarized using frequencies and percentages. Kaplan-Meier survival analysis was used to estimate survival probabilities at months 1, 3, 6, 9, and 12. The Kaplan-Meier estimator measures the level of survival at each time-period and imputes a probability of survival to the end of the period, given that the client began the period. Clients lost before the beginning of the period are not considered in the computed survival probability. Time to outcome event was censored due to loss to follow-up or non-occurrence of outcome event before the end of the study period. We then regressed each predictor variable against the outcome at 12-month retention to determine factors associated with retention. Factors with a p-value of <0.2 were used to build the general multivariable Cox regression model and elimination method of non-significant factors starting with those with higher p-values used to determine the final model. Adjusted hazard ratios (aHR) and corresponding 95% confidence intervals (95%CI) were computed and summarized.

### Ethical considerations

The study was submitted to and obtained approval from the Makerere University College of Health Sciences School of Public Health Higher Degrees Research and Ethics Committee (HDREC), and additional ethical clearance/approval from the Rwanda National Ethics Committee (RNEC) as study Institutional Review Boards (IRBs). Our study was retrospective in nature, using secondary data from patient files and registers hence no informed consent from individual clients was required. Instead, we used IRB approvals to seek administrative clearances from the facility in-charges of the various study sites to access client data.

### Inclusivity in global research

Additional information regarding the ethical, cultural, and scientific considerations specific to inclusivity in global research is included in S1 File.

## Results

**Fig 1:**
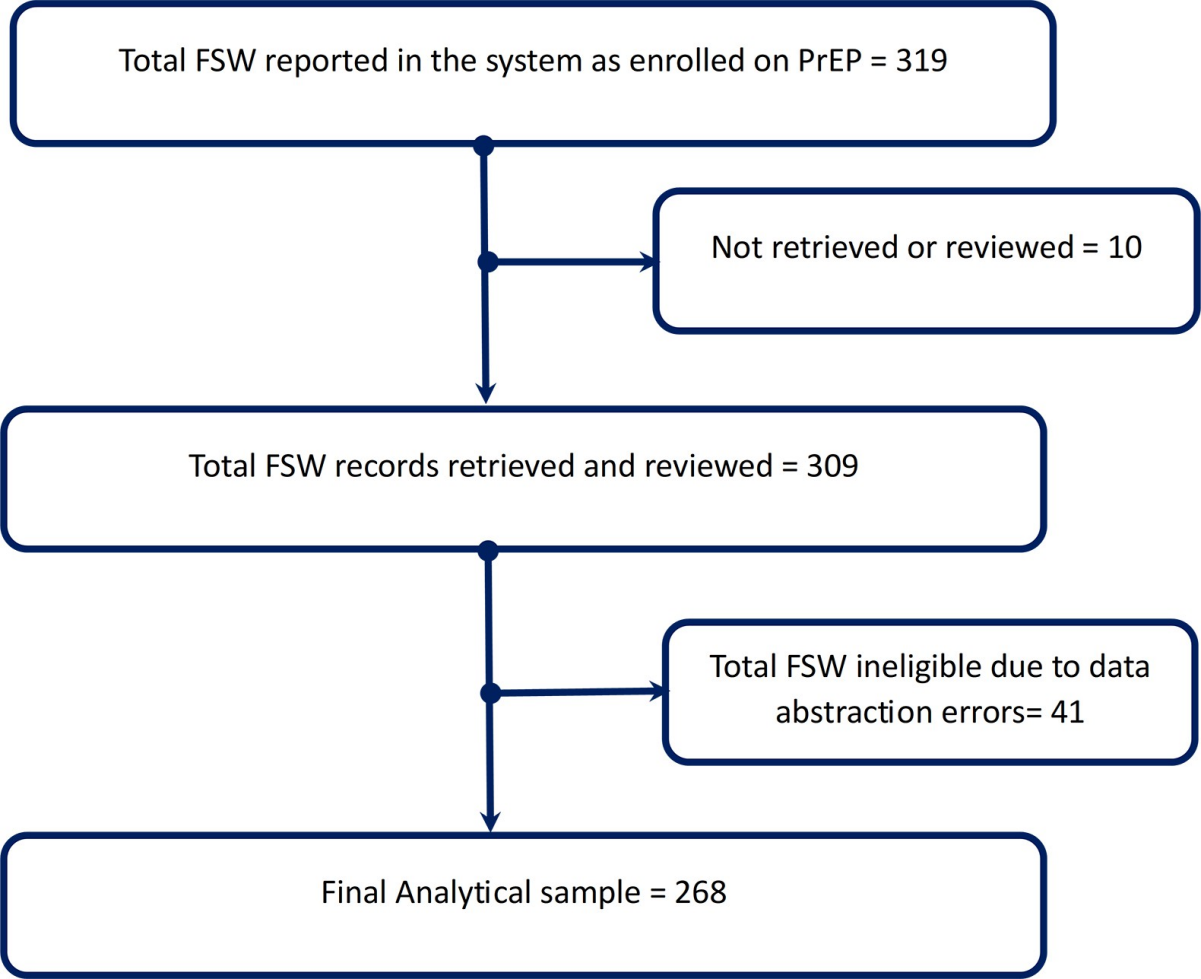
Analytical sample flow diagram.

From the PEPFAR reporting system (DATIM), 319 FSW were reported as enrolled on PrEP from the 5 health centers, although only 309 records were retrievable. Due to abstraction errors, 41 ineligible FSW were dropped. We thus remained with 268 FSW that made our analytical sample as shown by Fig 1. Each client’s visit was considered as an individual observation translating to 1,340 observations for the 268 FSW.

### Baseline characteristics of respondents

One half (50%, n = 133) of the respondents were aged 25–34 years, followed by those 35 years and above (36%, n = 95), while 15% (n = 40) were in the age bracket of 18–24 years. Forty-three percent (n = 115) of the respondents were from Kicukiro district, followed by those from Gasabo district (39%, n = 105), while eighteen percent (n = 47) of respondents were enrolled from Nyarugenge district (see Table 1).

**Table 1:** Baseline characteristics and PrEP survival probabilities of FSW at different follow-up time points.

Characteristic		Baseline		Retention at M1		Retention at M3		Retention at M6		Retention at M9		Retention at M12	
		N = 268	Col (%)	n	^1^Survival Prob. M1 (%)	n	Survival Prob. M3 (%)	n	Survival Prob. M6 (%)	n	Survival Prob. M9 (%)	n	Survival Prob. M12 (%)
**Overall Retention**		268	100	216	80.6	193	72.0	179	66.8	157	58.6	143	53.4
**Age-Group**													
	18–24	40	14.9	28	70.0	23	57.5	19	47.5	18	45.0	17	42.5
	25–34	133	49.6	110	82.7	97	72.9	93	69.9	85	63.9	79	59.4
	35+	95	35.5	78	82.1	73	76.8	67	70.5	54	56.8	47	49.5
**District**													
	Kicukiro	115	43.1	110	87.0	95	82.6	91	79.1	83	72.2	77	67.0
	Gasabo	105	39.3	90	85.7	79	75.2	71	67.6	63	60.0	59	56.2
	Nyarugenge	47	17.6	26	55.3	19	40.4	17	36.2	11	23.4	7	14.9
**Ever Married**													
	No	136	50.7	104	76.5	88	64.7	80	58.8	68	50.0	62	45.6
	Yes	132	49.3	112	84.9	105	79.6	99	75.0	89	76.4	81	61.4
**Highest Education Level attained**													
	None	42	15.7	33	78.6	31	73.8	30	71.4	28	66.7	25	59.5
	Primary School	196	73.1	159	81.1	142	72.5	130	57.1	112	57.1	103	52.6
	Secondary School	30	11.2	24	80.0	20	66.7	19	63.3	17	56.7	15	50.0
**Paid job besides sex work**													
	No	184	68.7	153	83.2	136	73.9	126	68.5	115	62.5	105	57.1
	Yes	84	31.3	63	75.0	57	67.9	53	63.1	42	50.0	38	45.2
**Living Alone**													
	Yes	236	88.1	193	81.8	172	72.9	161	68.2	139	58.9	125	53.0
	No	32	11.9	23	71.9	21	65.6	21	56.3	18	56.3	18	56.3
**Use of condom with vaginal sex in last7 days**													
	Always	54	20.2	49	90.7	46	85.2	46	85.2	43	79.6	41	75.9
	Never	38	14.2	26	68.4	22	57.9	21	55.3	19	50.0	19	50.0
	Sometimes	175	65.5	140	80.0	124	70.9	111	63.4	94	53.7	82	48.9
**Number of children <18 years**													
	0	36	13.4	25	69.4	22	61.1	20	55.6	20	55.6	18	50.0
	1–2	164	61.2	138	84.2	123	75.0	115	70.1	101	61.6	93	56.7
	3+	68	25.4	53	77.9	48	70.6	44	64.7	36	52.9	32	47.1
**More children wanted**													
	0	141	52.6	109	77.3	94	66.7	87	61.7	71	50.4	64	45.4
	1	47	17.5	39	83.0	34	72.3	32	68.1	26	55.3	23	48.9
	2+	80	29.9	68	85.0	65	81.3	65	75.0	60	75.0	56	70.0
**Methods to delay/avoid pregnancy**													
	Condoms	23	8.6	14	60.9	11	47.8	10	43.5	9	39.1	8	34.8
	None	41	17.2	34	82.9	31	75.6	30	73.2	26	63.4	24	58.5
	Hormonal	174	64.9	143	82.2	129	74.1	121	69.5	105	60.3	96	55.2
	Others	30	11.2	25	83.3	22	73.3	18	60.0	17	56.7	15	50.0

^1^ Survival Prob. M1 = Survival Probability at Month 1 in Percentages.

Slightly more than half of the respondents (52%, n = 136) had never been married before. Nearly three-quarters (73%, n = 196) of the respondents had completed primary education, eleven percent (n = 30) completed secondary education, while sixteen percent (n = 42) had never been to school. Sixty-nine percent (n = 184) of respondents had no formal employment and majority (88%, n = 236) reported to be living alone (see Table 1).

### Retention rates of FSW on PrEP

**Fig 2:**
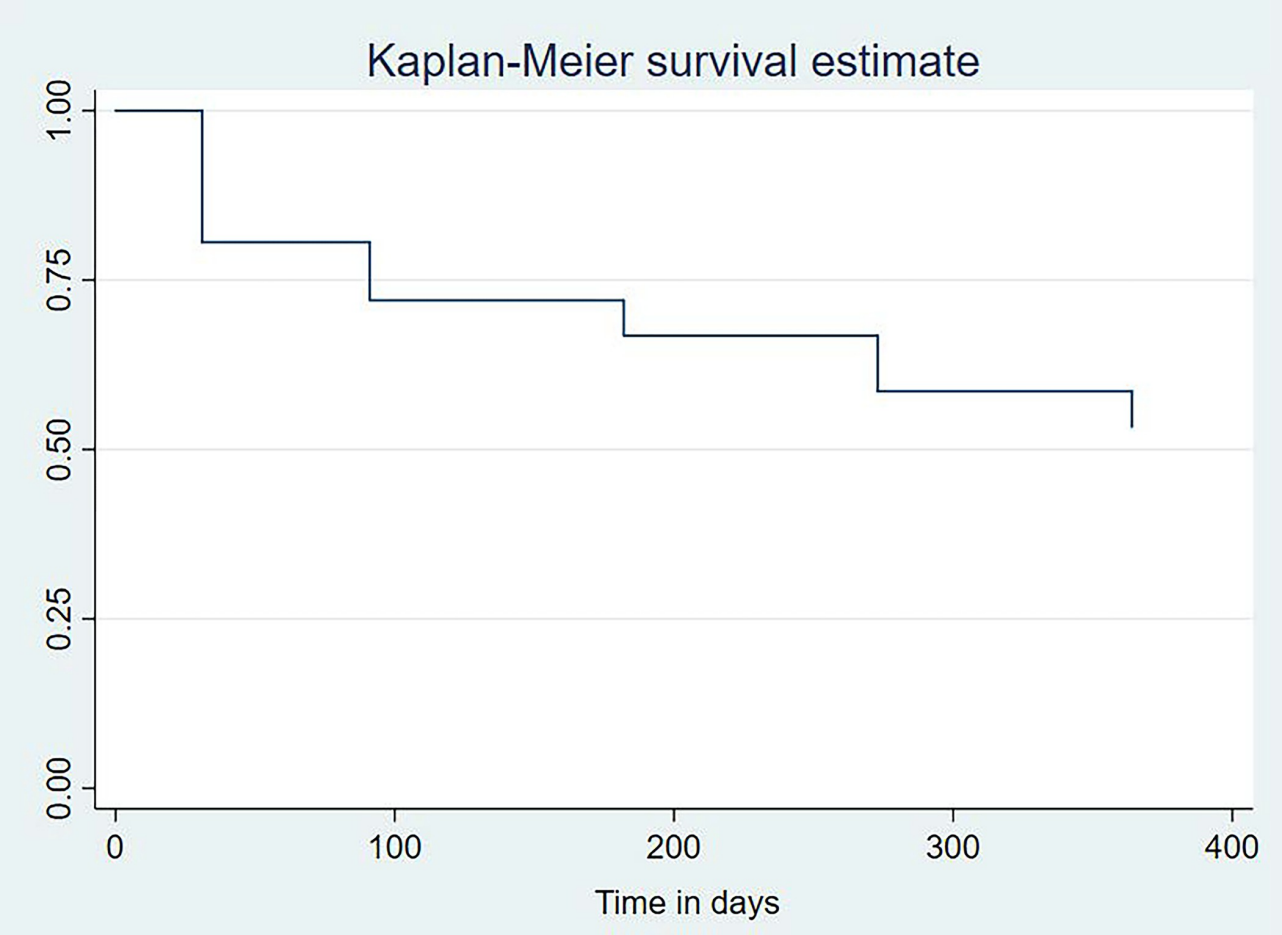
Kaplan-Meier survival probability for FSW initiated on PrEP in Kigali, Rwanda.

Overall, retention was 81% at month 1, dropping to 67% at month 6, and was at 53% by month 12 of follow-up. The Kaplan-Meier survival probability estimate graph depicted by Fig 2 shows that the biggest risk of dropping off PrEP was at month 1, followed by a continuous albeit steady decrease in retention rates at subsequent follow-up periods (months 3, 6, 9, and 12). PrEP dropout rates were 228, 65, 29, 49, and 36 per 100-persons years at months 1, 3, 6, 9 and 12 respectively.

Survival probability on PrEP decreased over the 12-month period of the study and was spread differently as depicted by Table 1. At month 1 of follow-up, all baseline characteristics had a PrEP survival probability of 50% and above. By month 6, the following characteristics had a survival probability on PrEP of less than 50%: FSW aged 18–24 years (M6 survival probability = 47.5%), FSW from Nyarugenge district (M6 survival probability = 36.2%), and FSW that used condoms as a method of contraception (M6 survival probability = 43.5%). By month-12 of follow-up, FSW that had never married before (M12 survival probability = 45.6%), those that had a paid job besides sex work (M12 survival probability = 45.2%), FSW who inconsistently use condoms for each sexual act (M12 survival probability = 48.9%), FSW with 3 or more children (M12 survival probability = 47.1%), and FSW that didn’t want to have any more children (M12 survival probability = 45.4%) or those that wanted to have only one (1) additional child (M12 survival probability = 48.9%), had joined the tally of those with PrEP survival probability of less than 50%.

Table 2 shows that FSW that wished to have 2 or more children demonstrated increased chances of being retained on PrEP than those that did not want to have any additional children (adjusted Hazard Ratio [aHR] = 1.654; 95%CI: 1.008, 2.713). Also, compared to those that opted for condoms as a method of contraception, FSW using hormonal method (aHR = 2.091, 95%CI: 1.181, 3.702) or those that didn’t use any method of contraception (aHR = 2.036, 95%CI: 1.006, 4.119) were more likely to be retained on PrEP. On the other hand, compared to those from Kicukiro district, FSW from Nyarugenge were less likely to be retained on PrEP (aHR = 0.290; 95%CI: 0.183, 0.458). Similarly, FSW that never used condoms (aHR = 0.329; 95%CI: 0.149, 0.726) or those that inconsistently used them (aHR = 0.413; 95%CI: 0.228, 0.749), were less likely to be retained on PrEP compared to those that consistently used condoms.

**Table 2:** Frequencies and hazard ratios of PrEP retention associated with covariates among FSW in Kigali.

	**Characteristic**	**Baseline (N = 268)**	**Retained at M12**	**Survival prob. at M12 (%)**	**Follow-up time PYO**	**Unadjusted**		**Adjusted Model**	
						**Crude Hazard Ratio**	**95%CI**	**aHR**	**95%CI**
**Age-Group**									
	18–24	40	17	42.5	23.02	1.000			
	25–34	133	79	59.4	98.21	**1.658**	**(1.041–2.641)**	-	-
	35+	95	47	49.5	69.45	1.323	(0.833–2.099)	-	-
**District**									
	Kicukiro	115	77	67.0	93.53	1.000			
	Gasabo	105	59	56.2	77.03	0.703	(0.467–1.059)	1.005	(0.632–1.598)
	Nyarugenge	47	7	14.9	20.04	**0.257**	**(0.173–0.382)**	**0.290**	**(0.183–0.458)**
**Ever married**									
	No	136	62	45.6	87.73	1.000			
	Yes	132	81	61.4	102.95	**1.587**	**(1.138–2.213)**	-	-
**Highest Education Level attained**									
	None	42	25	59.5	31.27	1.000			
	Primary School	196	103	52.6	138.90	0.830	(0.508–1.356)	-	-
	Secondary School	30	15	50.0	20.51	0.771	(0.402–1.479)	-	-
**Paid job besides sex work**									
	No	184	105	57.1	135.14	1.000			
	Yes	84	38	45.2	55.54	0.984	(0.402–2.410)	-	-
**Living alone**									
	No	32	18	56.3	20.77	1.000			
	Yes	236	125	53.0	169.91	0.733	(0.525–1.023)	-	-
**Use of condom with vaginal sex in last7 days**									
	Always	54	41	75.9	46.43	1.000			
	Never	38	19	50.0	23.02	**0.383**	**(0.191–0.765)**	**0.329**	**(0.149–0.726)**
	Sometimes	175	82	46.9	120.23	**0.390**	**(0.223–0.683)**	**0.413**	**(0.228–0.749)**
**Number of children <18 years**									
	0	36	18	50.0	22.69	1.000			
	1–2	164	93	56.7	121.46	1.287	(0.781–2.120)	-	-
	3+	62	32	51.6	46.53	0.999	(0.583–1.711)	-	-
**More children wanted**									
	0	141	64	45.4	92.98	1.000			
	1	47	23	48.9	33.43	1.124	(0.745–1.695)	1.065	(0.651–1.741)
	2+	68	32	47.1	64.27	**2.058**	**(1.321–3.206)**	**1.654**	**(1.008–2.713)**
**Methods to delay/avoid pregnancy**									
	Condoms	23	8	34.8	11.77	1.000			
	None	41	24	58.5	30.85	**1.997**	**(1.042–3.828)**	**2.036**	**(1.006–4.119)**
	Hormonal	174	96	55.2	127.14	**1.812**	**(1.082–3.037)**	**2.091**	**(1.181–3.702)**
	Others	30	15	50.0	20.93	1.575	(0.814–3.048)	1.530	(0.727–3.222)

## Discussion

This study on retention on PrEP among FSW in Kigali has demonstrated that retention rates are higher in the immediate period following enrolment but decreased over time. The 12-month retention of 53% observed in this study is close to that of 47% obtained in the demonstration project among FSW in Benin and that of 55% seen in the HPTN082 trial among AGYW [15, 35]. It is however, higher than the 12-month retention of 22% seen in the TAPS study in South Africa and that seen in the San Francisco Primary care clinics of 38% [16, 36]. On the other hand, this 12-month retention is lower than the 73% seen in the PrEP demonstration project of FSW in Senegal and the 90% seen in the Indian study among FSW [17, 37]. We believe that the 12-month retention was positively influenced by the follow-up offered by the FSW peer navigators as in other studies with good retention.

In this study, 81% of FSW were retained by month-1 follow-up. This is similar to the 84% early retention seen in the San Francisco study but lower than that seen in the Senegalese study where retention at month-1 follow-up was 90% [36, 37]. The sharpest decrease in retention happened in the first 31 days, with a dropout rate of 228 per 100-person years being registered compared to the rate of 36 per 100-person years at month-12: implying that the likelihood of being retained on PrEP improved as the FSW stayed longer on the program. This finding is similar to that seen in the FSW community study in India [17]; stressing the importance of being vigilant in obtaining commitment to PrEP in the early days of PrEP initiation.

Findings from this study point to the fact that the desire to have 2 or more children was a motivator to be retained on PrEP. The desire to stay healthy HIV free and bare HIV negative children has been noted as motivator to be retained on PrEP [38].Relatedly, the desire to stay healthy and maintain earning potential so as to take care of their children has also been cited as a motivator to being retained on PrEP [39]. This further emphasizes the need to integrate PrEP into sexual and reproductive health services [40]. FSW on PrEP that come to clinics for contraceptive counseling or for their next dose of injectable contraceptives get an opportunity to hear PrEP reinforcing messages [39]. The additional messages provide an advantage for PrEP retention when compared to FSW who access their condoms for contraception without interacting with a health care provider.

The debate on whether PrEP users continue to use condoms consistently is one that rages on with some evidence showing laxity in condom use among PrEP users [39]. This is especially the case with clients paying more money for condomless sex [41]. On the other hand, a study in Kenya demonstrated increased condom use among FSW enrolled on PrEP [42]. Nonetheless, for FSW that show a laxed attitude towards consistent condom use, this laxity can be extended to other health matters even affecting PrEP retention, because of their distorted risk perception or other socioeconomic pressures.

Being in an urban area comes with a woven web of both challenges and opportunities. Results in this study show that FSW from Nyarugenge, which is the central business district, had poor retention compared to those from Gasabo and Kicukiro district. This is consistent with other study findings given the challenges experienced by urban dwellers when accessing health care. These include frequent relocations, cost of transport to and from clinics, or failure to remember appointments because they are busy [43]. This negatively affects PrEP retention as demonstrated in this study. An ultra-urban clinic will thus need a robust follow-up and tracking mechanism for its FSW enrolled on PrEP.

## Study limitations and strengths

Our study used secondary data from KP files in which some data errors were found. We worked with the health center KP focal persons to correct any obvious transcription errors which led to improvements in data quality. Not all factors that could be potentially associated with PrEP retention were collected and availed for analysis. These includes factors such as drug side effects, problematic dosing schedules, decreased risk perception, relocation of FSW, stigma, exacerbation of pre-existing condition, disclosure concerns, positive interaction with PrEP providers, etc., [44–46]. Instead, we were only able to analyze the data abstracted from the health facility KP files. Our study considered every FSW enrolled at the base period of April-July 2020 hence limiting any selection bias. Our study censored PrEP clients that didn’t return for follow-up within 14 days past their appointment date, limiting our ability to estimate those who reinitiated PrEP after this period.

## Conclusions

PrEP retention rates were highest in the early days of PrEP initiation, but by 12 months of follow-up, only just over one half (53%) of FSW were retained on PrEP. FSW that desired to have more children, and those using hormonal or not using contraception had higher likelihoods of retention on PrEP while those from urban clinics and those that inconsistently or never used condoms were less likely to be retained on PrEP. Program Managers need to be vigilant at eliciting early commitment to PrEP. In addition, PrEP retention monitoring needs to be directed towards FSW who never or inconsistently use condoms for each sexual act as their distorted risk perception is likely to negatively affect PrEP retention. Creating flexi-hours for refills and establishing mechanisms for delivering PrEP for clients at their convenience are key in addressing high dropout rates for clients enrolled at urban clinics.

## Supporting information

S1 File Inclusivity in global research questionnaire. (DOCX)
